# C-fibers may modulate adjacent Aδ-fibers through axon-axon CGRP signaling at nodes of Ranvier in the trigeminal system

**DOI:** 10.1186/s10194-019-1055-3

**Published:** 2019-11-12

**Authors:** Jacob C. A. Edvinsson, Karin Warfvinge, Diana N. Krause, Frank W. Blixt, Majid Sheykhzade, Lars Edvinsson, Kristian A. Haanes

**Affiliations:** 1 Department of Clinical Experimental Research, Copenhagen University Hospital, Rigshospitalet-Glostrup, Copenhagen, Denmark; 2 Department of Clinical Sciences, Division of Experimental Vascular Research, Lund University, Lund, Sweden; 3 Department of Pharmacology, School of Medicine, University of California at Irvine, Irvine, CA USA; 4 Department of Drug Design and Pharmacology, Faculty of Health and Medical Sciences, |University of Copenhagen, Copenhagen, Denmark; 5 Department of Neurology, McGovern Medical School, University of Texas Health Science Center at Houston, Houston, TX USA

**Keywords:** CGRP, Node of Ranvier,, Aδ-fibers, C-fibers, Trigeminal ganglion, CASPR

## Abstract

**Background:**

Monoclonal antibodies (mAbs) towards CGRP or the CGRP receptor show good prophylactic antimigraine efficacy. However, their site of action is still elusive. Due to lack of passage of mAbs across the blood-brain barrier the trigeminal system has been suggested a possible site of action because it lacks blood-brain barrier and hence is available to circulating molecules. The trigeminal ganglion (TG) harbors two types of neurons; half of which store CGRP and the rest that express CGRP receptor elements (CLR/RAMP1).

**Methods:**

With specific immunohistochemistry methods, we demonstrated the localization of CGRP, CLR, RAMP1, and their locations related to expression of the paranodal marker contactin-associated protein 1 (CASPR). Furthermore, we studied functional CGRP release separately from the neuron soma and the part with only nerve fibers of the trigeminal ganglion, using an enzyme-linked immunosorbent assay.

**Results:**

Antibodies towards CGRP and CLR/RAMP1 bind to two different populations of neurons in the TG and are found in the C- and the myelinated Aδ-fibers, respectively, within the dura mater and in trigeminal ganglion (TG). CASPR staining revealed paranodal areas of the different myelinated fibers inhabiting the TG and dura mater. Double immunostaining with CASPR and RAMP1 or the functional CGRP receptor antibody (AA58) revealed co-localization of the two peptides in the paranodal region which suggests the presence of the CGRP-receptor. Double immunostaining with CGRP and CASPR revealed that thin C-fibers have CGRP-positive boutons which often localize in close proximity to the nodal areas of the CGRP-receptor positive Aδ-fibers. These boutons are pearl-like synaptic structures, and we show CGRP release from fibers dissociated from their neuronal bodies. In addition, we found that adjacent to the CGRP receptor localization in the node of Ranvier there was PKA immunoreactivity (kinase stimulated by cAMP), providing structural possibility to modify conduction activity within the Aδ-fibers.

**Conclusion:**

We observed a close relationship between the CGRP containing C-fibers and the Aδ-fibers containing the CGRP-receptor elements, suggesting a point of axon-axon interaction for the released CGRP and a site of action for gepants and the novel mAbs to alleviate migraine.

## Introduction

The first drugs aimed directly at interfering with a signaling pathway implicated in the pathophysiology of migraine were recently approved by the FDA (Food and Drug Administration) and EMA (European Medicines Agency). These drugs specifically target the calcitonin gene-related peptide (CGRP) signaling pathway [1, 2]. Although not all antimigraine drug candidates have made it to the clinic, all drugs that inhibit CGRP signaling have shown positive effects in all available trials to date. These include drugs aborting acute attacks with CGRP receptor antagonists (gepants) to prophylaxis against chronic or frequent episodic migraine with monoclonal antibodies (mAbs) against CGRP or the CGRP receptor [1, 3, 4]. The highly effective CGRP/CGRP receptor antibodies have not only given strong evidence for the involvement of CGRP in migraine pathophysiology, but in addition provided clues to understand the mechanisms behind a migraine attack. Since the mAbs i) do not cross the blood-brain barrier (BBB) [5], and ii) there is no evidence for a leaky BBB opening during an attack [6–8], the antimigraine target must reside outside the central nervous system (CNS).

Sensory neurons related to migraine pain are located in the first division of the trigeminal ganglion (TG) which is a part of the trigeminovascular system. The presence of a large population of CGRP neurons within the TG signifies a major role for CGRP in trigeminal transmission [1, 9]. Immunohistochemistry with antibodies against CGRP and in situ hybridization to localize CGRP mRNA have shown that approximately half of all neurons in the TG express CGRP [9]. CGRP is expressed in unmyelinated C-fiber sensory nerves while the CGRP receptor is expressed in myelinated Aδ-sensory nerves [9].

Recently Burstein and colleagues showed that cortical spreading depression induced dilation and plasma protein extravasation were unaffected by the anti-CGRP mAb, fremanezumab [10]. This study adds to their previous work, where they have postulated that the new anti-migraine drugs may act to prevent CGRP binding to trigeminal Aδ-fibers in the periphery [11]. Furthermore, with this in mind, we set out to further investigate putative sites of CGRP action in the trigeminovascular system, particularly in the TG, which we have hypothesized to be the “tuner” for trigeminal pain [1, 12]. Further, these interacting sites may be important for peripheral and/or central sensitization, which are key modulators of pain threshold in migraineurs. In vitro studies have shown that CGRP can acutely modulate excitation in the somas of TG [13, 14], but possible effects on the corresponding fibers have received little interest. We hypothesize that CGRP could modulate pain transmission through two pathways: (i) regulation of excitatory potentials and signaling in TG neuronal somas and/or (ii) modulation of conduction potentials in TG sensory nerve fibers.

Saltatory conduction in myelinated axons such as Aδ-sensory nerves involves distinct domains consisting of the nodes of Ranvier along with the internodal, the juxtaparanodal and the paranodal regions of the axon [15]. These domains arise from interactions between axons and myelinating glial cells, mainly Schwann cells in the peripheral nervous system (PNS) [16]. In addition, the domains are known to have different compositions of ion channels and therefore different physiological roles [17, 18], e.g. a higher concentration of voltage-gated sodium channels are localized to the nodal region while delayed rectifier potassium channels are mainly concentrated to the juxtaparanodal region as well as Schwann cells.

Contactin-associated protein 1 (CASPR) is uniquely localized to the paranodal regions during the onset of myelination and this protein is a reliable marker for axonal nodes [18, 19]. CASPR interacts with the glial adhesion molecule Neurofascin-155 to anchor the Schwann cell to the axon [18]. Therefore, we examined CGRP signaling elements with respect to localization of CASPR to test the hypothesis that nodes of Ranvier are a possible target for modulation of conduction speed or potential thresholds in trigeminal transmission.

Our study was designed to examine if there is possibility for crosstalk between sensory nerve fibers that is mediated by CGRP. With detailed neuroanatomical work using a palette of selective antibodies we provide evidence for a novel site of action on the Aδ-fibers for the new CGRP receptor mAbs and the gepants.

## Materials and methods

### Immunohistochemistry

Male Wistar rats (*n* = 8, 260–300 g), raised and maintained under standard laboratory conditions (12/12 h light-dark cycle, with dark beginning at 7 p.m.) were used in this study. The animals were housed 2–3 rats together in Tall IVC Rat Cages (Innovive) with chow (RM1, SDS) and water ad libitum*.* The experimental procedures were approved by the Lund University Animal Ethics Committee (M43–07) and performed in accordance with the European Community Council Directive on ‘The Protection of Animals Used for Scientific Purposes’ (2010/63/EU). The rats were anesthetized with CO_2_ and decapitated, whereupon the right and left TG where carefully removed as well as segments of dura mater. The dura mater segments were spread out on microscope slides (Superfrost, ThermoFisher), and allowed to dry for approximately 15 min.

The tissues were then fixated in 4% paraformaldehyde (Sigma, St Louis, USA) diluted in phosphate buffered saline (PBS) for 2–4 h. The fixated tissues were cryoprotected using first a 10% and then 25% sucrose (Sigma) in Sorensen’s phosphate buffer overnight. Following this, the TG was embedded in a gelatin medium (30% egg albumin, 3% gelatin, Sigma) and subsequently cryosectioned at 10 μm and stored at − 20 °C until use. The dura mater slides were then stored in − 20 °C after cryoprotection (for treatment of whole mounts, see [20]).

The TG sections and dura mater slides where allowed to thaw in room temperature and subsequently rehydrated and permeabilized in 0,25% Triton X-100 diluted in PBS (PBS-T; Sigma) for 2 × 15 minutes. Primary antibodies diluted in PBS-T containing 1% bovine serum albumin (BSA; Sigma) were applied to the sections that were then incubated at + 4 °C overnight. Sections were subsequently rinsed of excess antibodies in PBS-T for 2 × 15 min. The sections were then incubated with secondary antibodies diluted in PBS-T for 1 h in a dark room (details on antibodies can be found in Additional file 3 Table S1 and Additional file 4 Table S2). Lastly, excess secondary antibodies were rinsed with PBS-T 2 × 15 min and the specimens mounted with anti-fading medium (Vectashield, Vector laboratories, Burlingame, CA, USA). The process was repeated with an additional primary and secondary antibody before mounting when performing double immunohistochemistry. Negative controls followed the same procedure but in the absence of primary antibodies.

Examination of the sections were performed using an epifluorescence microscope (Nikon 80i, Tokyo, Japan) coupled to a Nikon DS-2 MV camera. Images were obtained using NIS basic research software (Nikon, Japan).

### CGRP release

Six (*n* = 6) additional rats were anaesthetized by CO_2_ inhalation and decapitated. These rats were housed in Eurostandard cages (Type VI with 123-Lid) 2–6 together. The Danish Animal Experimentation Inspectorate approved all procedures. The protocol is described in detail elsewhere [21, 22]. TGs were dissected and immersed in 10 ml synthetic interstitial fluid (SIF, composition: 108 mM NaCl, 3.5 mM KCl, 3.5 mM MgSO_4_, 26 mM NaHCO_3_, NaH_2_PO_4_, 1.5 mM CaCl_2_, 9.6 mM NaGluconate, 5.6 mM glucose and 7.6 mM sucrose; pH 7.4.) at 37 °C for 30 min.

TGs were randomized, placed in Eppendorf tubes in a heating block at + 37 °C. TGs were washed five times (each wash 10 min), with 300 μl SIF. After 10 min incubation with 300 μl SIF, 200 μl samples for measuring the basal CGRP release were collected from the tissues, mixed with 50 μl enzyme immunoassay buffer (containing protease inhibitors) and stored at − 20 °C until analysis, within a week after the experiment was performed. The release of CGRP from TGs with soma and TGs without soma was induced by 60 mM K^+^-SIF. To maintain equal osmolarity, Na^+^ was replaced with an equimolar amount of K^+^ when making the depolarizing buffer. Experiments by others have shown that 10 min incubation is sufficient for a significant and reproducible release of CGRP over basal levels (19). CGRP release data point from the TG was excluded as the CGRP baseline was 0 pg/ml.

The samples were processed using a commercial EIA kit, Human CGRP ELISA KIT (SPIbio, Paris, France) to study CGRP release. The protocol was performed following the manufacturer’s instructions and the optical density was measured at 410 nm using a micro-plate photometer (Tecan, Infinite M200, software SW Magellan v.6.3, Männedorf, Switzerland).

All quantitative data were analyzed using GraphPad 8.0, and are presented as mean ± SEM. The n indicates the number of animals. The difference between the variables were compared with a two-sided paired Student’s t-test, the data past the Shapiro-Wilks test for normality. For art work, Servier Medical Art by Servier under a Creative Commons 3.0 license was used.

## Results

### CGRP release from fiber-rich regions of the TG

**Fig. 1:**
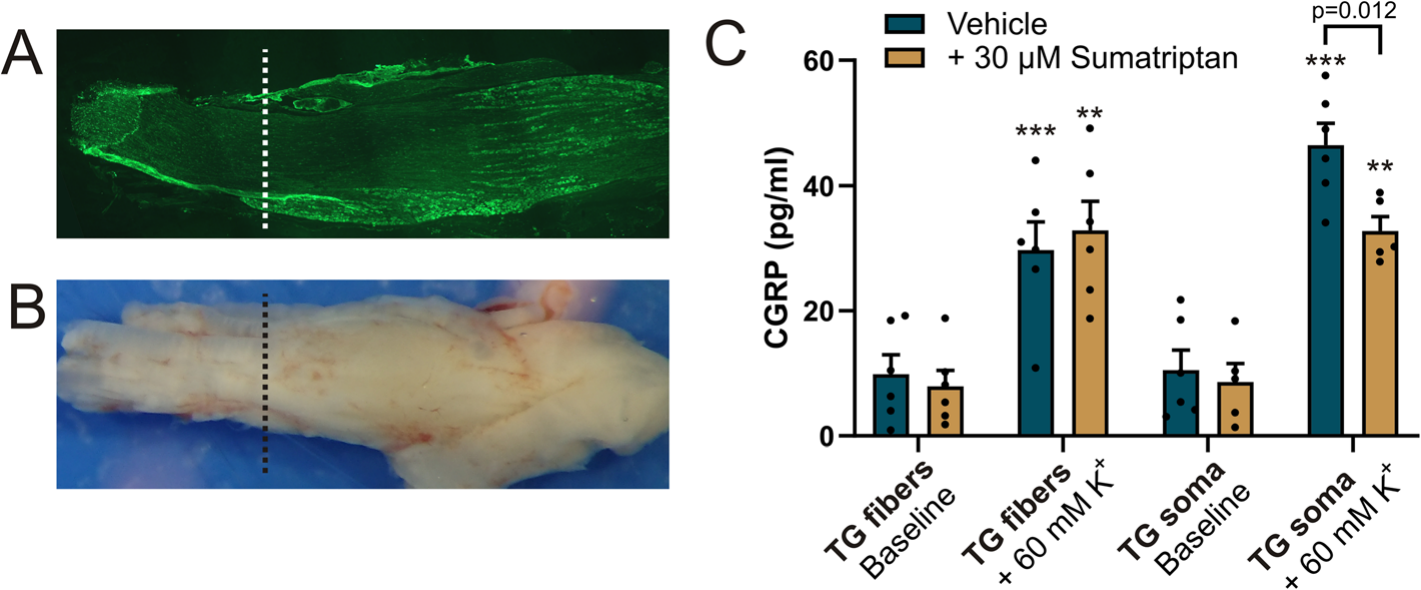
CGRP release in vitro from two different regions isolated from the TG. The soma are located in the center of the TG (**a**). **b** The TG was cut into one area that which is fiber-rich and devoid of neuronal cell bodies (“TG fibers”) and the other that was rich in neuronal somas (“TG soma”). CGRP was increased significantly from baseline in all samples stimulated with 60 mM K^+^ (** *p* > 0.01, *** *p* < 0.001, paired student’s T-test). Sumatriptan significantly inhibited the CGRP release only from the from TG containing soma. Data is expressed as mean ± SEM, *n* = 6 for vehicle, *n* = 5 for sumatriptan

In the TG, CGRP-containing C-fibers often run parallel to Aδ-fibers that contain CGRP receptor elements [9]. Thus, we hypothesized that CGRP may have important modulatory effects on the nerve fibers. In order for such a hypothesis to be relevant, we needed to confirm that CGRP could actually be released from the fiber itself and not just from the neuronal soma. We therefore divided the TG in two parts, one containing only nerve fibers and one part containing soma with nerve fibers (Fig. 1). Using a depolarization stimulus (60 mM K^+^-SIF), we induced CGRP release from the nerve fibers themselves (29.7 ± 4.5 pg/mL) as well as from the TG region containing somas (46.4 ± 3.5 pg/mL). Interestingly, only CGRP release from the somas could be inhibited with sumatriptan (from 46.4 ± 3.5 pg/mL to 32.8 ± 2.3 pg/mL, *p* = 0.012). No inhibition was seen in the nerve fiber segment (from 29.7 ± 4.5 pg/mL to 32.9 ± 4.6 pg/mL, *p* = 0.63). Thus, we demonstrated that CGRP could be released from both segments of the TG, but this release could only be significantly inhibited with sumatriptan at the soma segment.

### Expression of CGRP receptors in nerve fibers

**Fig. 2:**
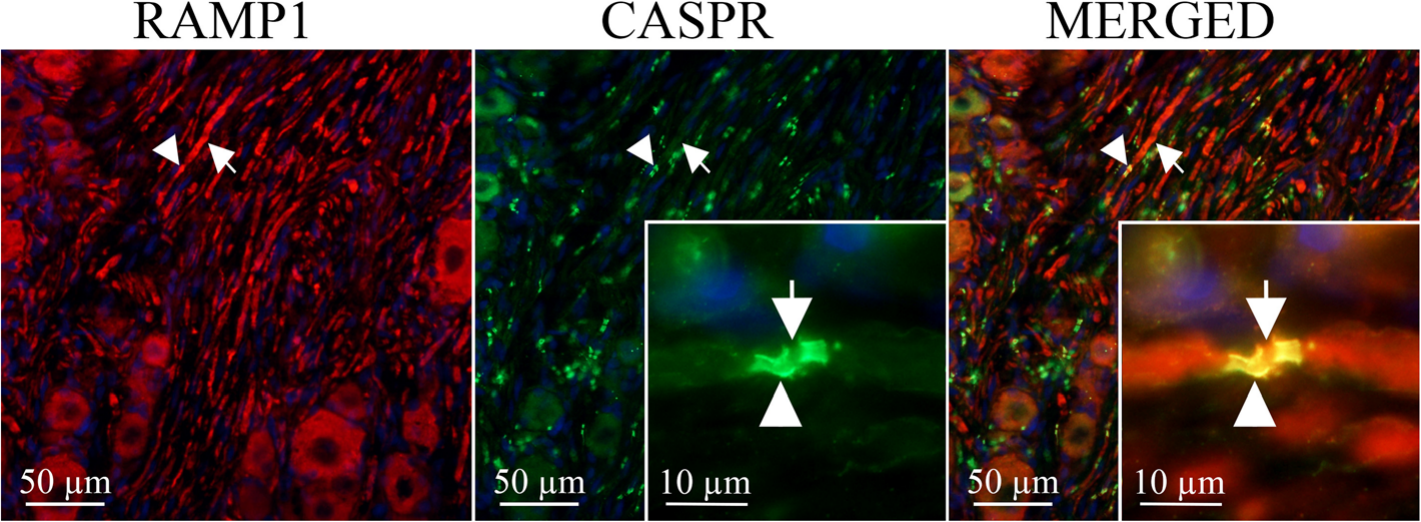
CGRP receptor localization at the paranodal region in the TGImmunohistochemical localization of the CGRP receptor protein RAMP1 (red, arrow) in myelinated axons of the trigeminal ganglion labeled with CASPR (green, arrowhead), a marker for nodes of Ranvier. Merged images reveal co-localization (yellow) of RAMP1 with CASPR at the nodes

CGRP receptors were localized in larger neurons and axonal fibers, consistent with myelinated Aδ sensory fibers, as we have previously reported [23]. Since we demonstrated that CGRP can be functionally released in a part of the TG that only contains fibers, we further examined the expression of CGRP receptor targets in trigeminal fibers. We used a polyclonal antibody to CASPR as an immunofluorescent marker for nodes of Ranvier in myelinated axons (Additional file 1 Figure 1**)**. CASPR immunoreactivity indicated a dense plexus of myelinated fibers within the ganglion (Fig. 2). CASPR-positive staining showed a characteristic punctate doublet pattern that reflects the location of this protein in the paranodal regions (stained) on either side of the actual node (unstained) [16, 18]. We then used an antibody to RAMP1 (an integral protein of the CGRP receptor) to visualize CGRP receptors in rat trigeminal ganglion (Fig. 2). Double staining revealed co-localization of RAMP1 and CASPR (Fig. 2) indicating CGRP receptors are discretely located in the paranodal region of myelinated trigeminal axons.

### Localization of CGRP receptors and PKA signaling in trigeminal axons

**Fig. 3:**
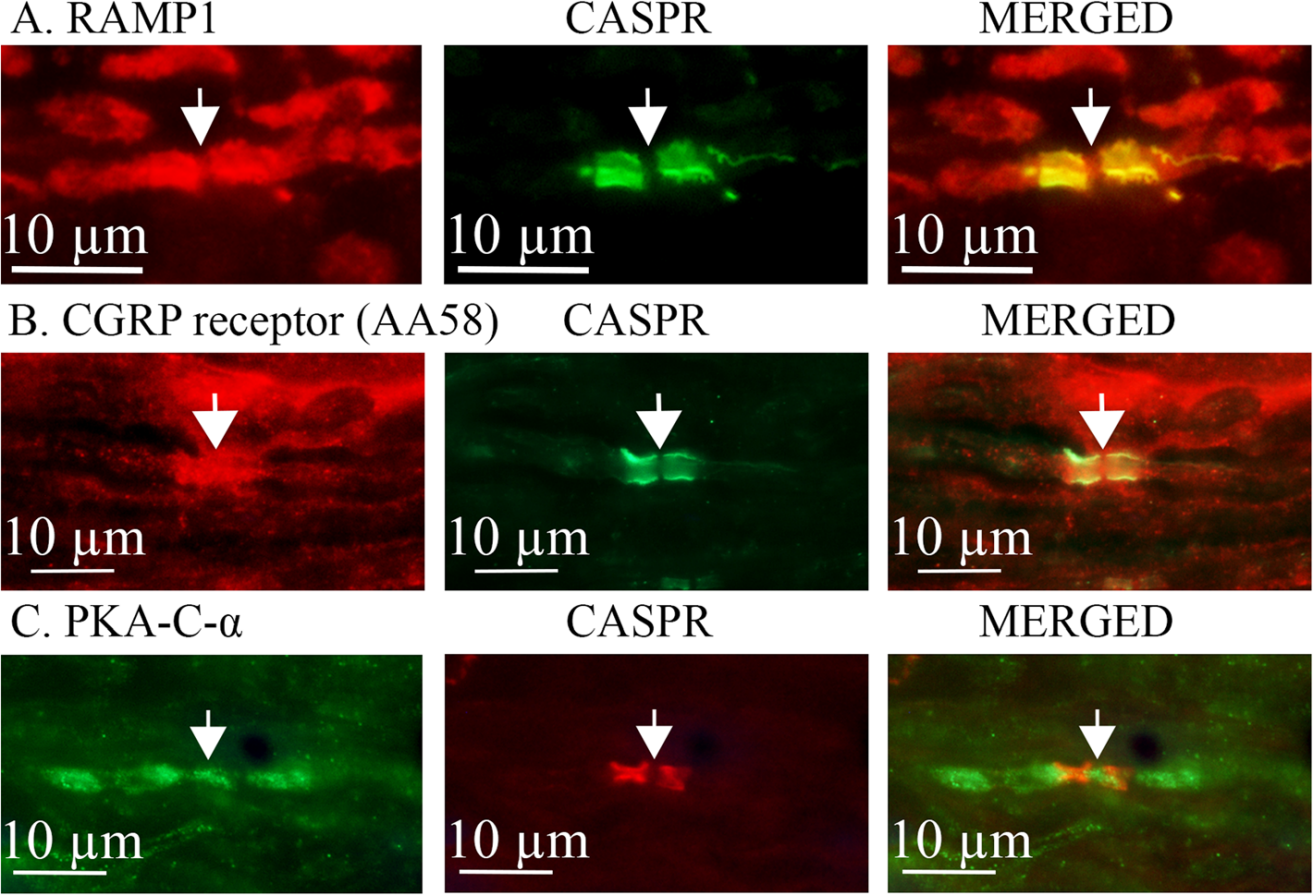
Localization of CGRP receptor and PKA at the nodes of Ranvier in trigeminal axons. CGRP receptors were labeled using a RAMP1 antibody (**a**) or the AA58 antibody targeted to the functional CGRP receptor complex (**b**). Both antibodies showed co-localization with CASPR. Evidence of relevant signaling pathways is indicated by positive staining for the catalytic subunit of Protein Kinase A (PKA) at the nodes (**c**). Arrows indicate the node of Ranvier

Both the RAMP1 antibody (Fig. 3a) and the AA58 antibody (targeted to the functional CGRP receptor complex [24], Fig. 3b), show that CGRP receptors specifically co-localize with CASPR, indicating the receptor is present in the paranodal region of the axon. Furthermore, the AA58 antibody revealed that the functional CGRP receptor was expressed in regions we suggest to be Schwann cells (Fig. 3b). CGRP receptors are G-protein coupled receptors classically linked to G_s_ and increases in cyclic AMP [25]. Evidence of relevant signaling pathways at the nodes was indicated by positive staining for the catalytic subunit of Protein Kinase A (PKA) which is activated by cAMP (Fig. 3c). Note, however, that while the PKA catalytic subunit α is found in the axon around the node region, it does not appear to co-localize with CASPR. This suggests different subcellular locations for the CGRP receptor and PKA within the axon [26], with the receptor and CASPR being anchored to the membrane whereas PKA is within the axon cytosol. This is similar to what was observed for cytosolic endosomes labeled with an Ab to EEA1 (early endosomal autoantigen 1) (Additional file 2 Figure S2).

### Relationship of CGRP-containing fibers with nodes on myelinated axons in the TG

**Fig. 4:**
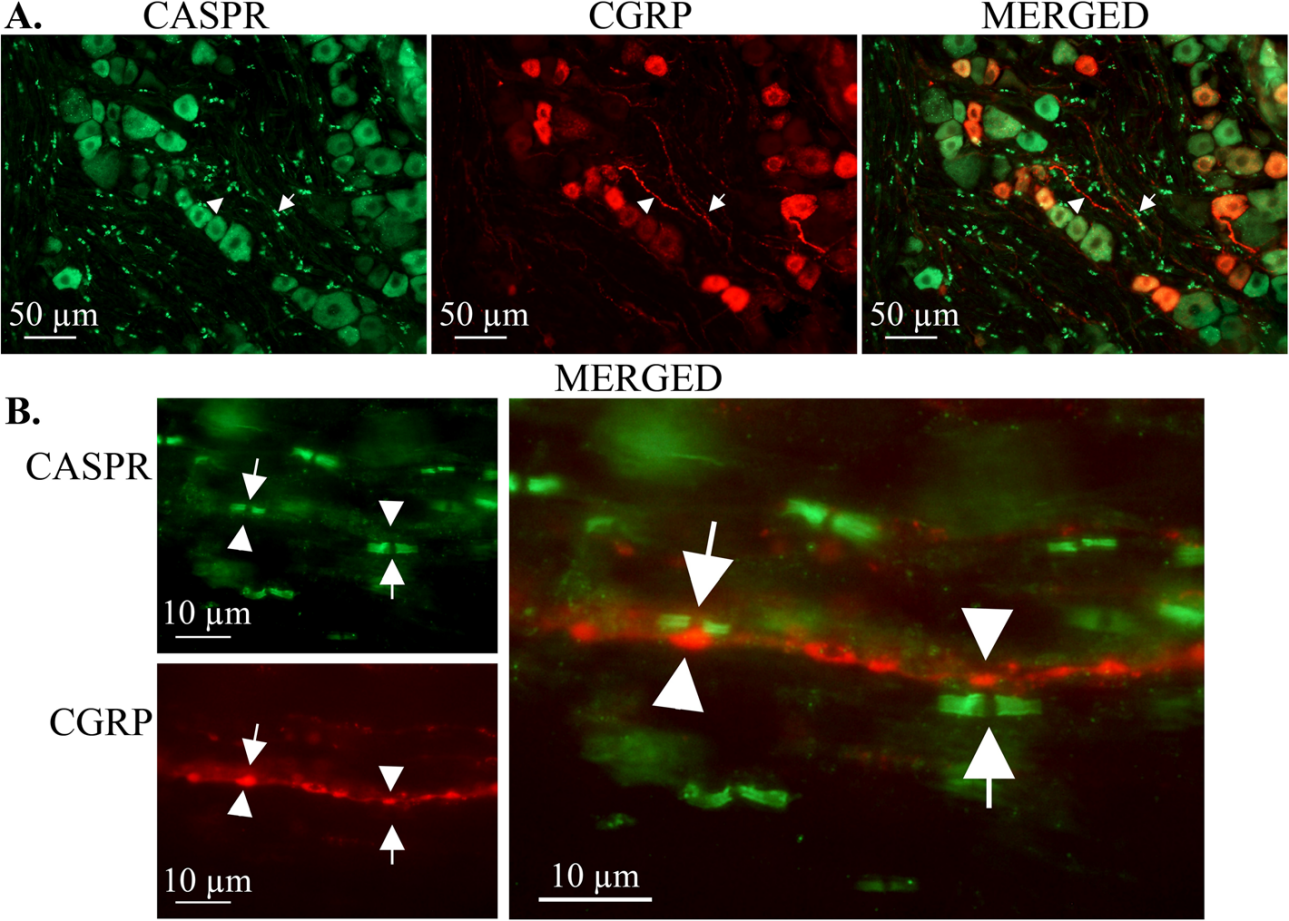
Immunolocalization of CGRP in rat trigeminal ganglia in relation to CASPR-positive axonal nodes. **a** The CGRP Ab labeled smaller neurons and thin fibers (arrowhead) within the ganglia. Double staining with the CASPR Ab (arrow) showed no co-localization (merged). **b** Higher magnification shows a CGRP-labeled axon in close proximity to CASPR-positive axons. CGRP-containing axonal varicosities (arrow), which are putative release sites, align directly across from CASPR-positive nodes of Ranvier (arrowhead) on the adjacent axon

CGRP immunoreactivity was observed in smaller neurons and thin C-fibers within the trigeminal ganglion. There was no co-localization of CGRP with the nodal marker CASPR. These findings are consistent with CGRP localization in unmyelinated C-fibers and their cell bodies and similar to what we have reported previously [23]. In the current study we observed that there is often a close association between the thin and thick sensory fibers (Fig. 4a). Higher magnifications of merged images of CGRP and CASPR immunofluorescence show CGRP-containing axonal varicosities, which are putative release sites, align directly across from CASPR-positive nodes of Ranvier along the adjacent myelinated axon (Fig. 4b). These remarkable images suggest that the node of Ranvier is a site of axon-axon communication where CGRP released from C-fiber varicosities act on CGRP receptors located within the nodes of Aδ-sensory nerves.

### Localization of 5-hydroxytryptamine receptors in the TG

**Fig. 5:**
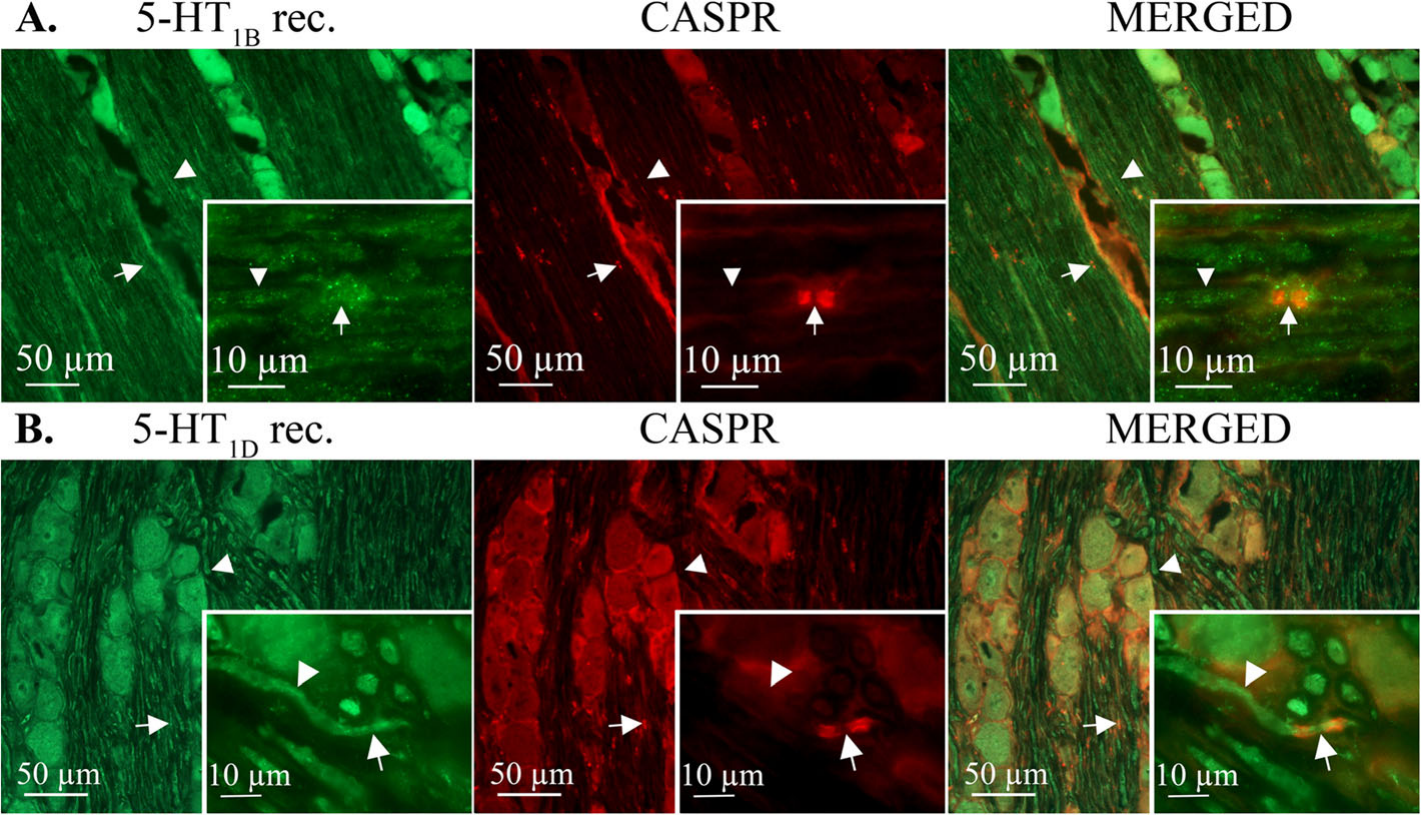
Immunolocalization of 5-HT_1B_ and 5-HT_1D_ receptors in the TG. Green positive immunofluorescence for 5-HT_1B_ (**a**) and 5-HT_1D_ (**b**) receptors throughout the Aδ-fiber, also at the node of Ranvier (monoclonal CASPR, red, arrow) No clear co-localization with CASPR was detected. Arrowheads point out immunopositive axons for both receptors

Since the triptans have proven successful as antimigraine medication, we also investigated localization of the 5-hydroxytryptamine (5-HT) receptors 5-HT_1B_ and 5-HT_1D_ in TG neurons and fibers in relation to CASPR (Fig. 5). In contrast to the CGRP receptor, neither 5-HT_1B_ nor 5-HT_1D_ receptors showed specific co-localization with CASPR. Immunoreactivity for 5-HT_1B_ and 5-HT_1D_ was observed in TG axons. 5-HT_1B_ and 5-HT_1D_ receptor immunoreactivities were also observed in neuronal soma, satellite glial cells and Schwann cells. These data match the results from the functional release, as sumatriptan only inhibited CGRP release when the TG preparation contained neuronal bodies (Fig. 1).

### Peripheral nerve fibers in the dura mater

**Fig. 6:**
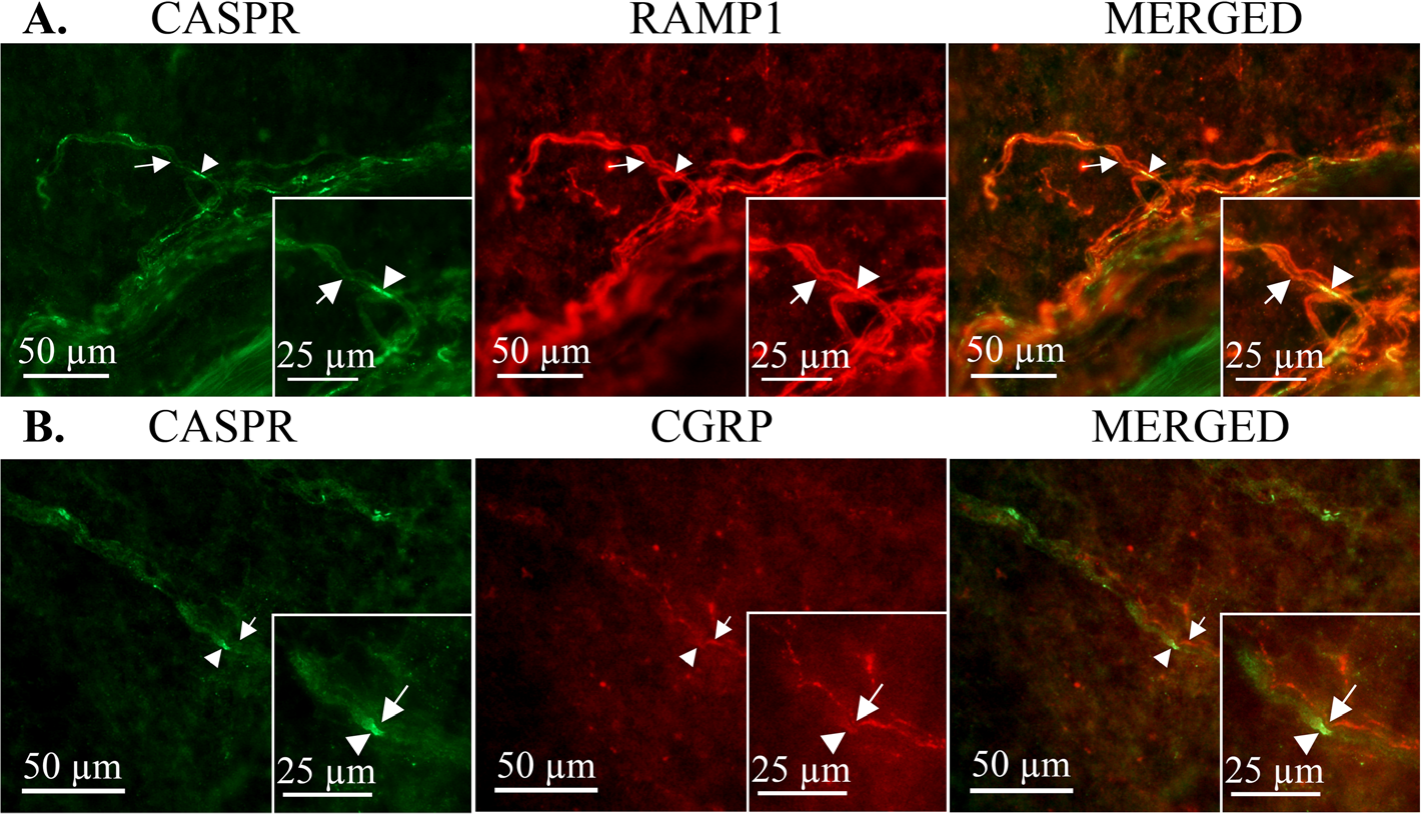
Expression of RAMP1 and CGRP in relation to the node of Ranvier in rat dura mater spreads. RAMP1 (**a**) and CGRP (**b**) had similar localization at the node of Ranvier in the rat dura mater as for the TG. Top; CASPR (green, arrowhead), RAMP1 (red, arrow). Bottom; CASPR (green, arrowhead), CGRP (red, arrow)

Since peripheral trigeminal afferents innervate the dura mater, we examined the sensory nerves in this tissue. Also in this preparation, the RAMP1 protein of the CGRP receptor complex co-localized with CASPR (Fig. 6a), further supporting a nodal location of the receptor. Sensory C- and Aδ-fibers characteristically run together. Similar to what we found in the TG (Fig. 4), there was a close relationship between CASPR positive Aδ-fibers and CGRP positive C-fibers in the dura mater (Fig. 6b). These findings suggest that CGRP axon-axon signaling may occur along the entire trigeminal pathway, both within the ganglion and along afferent projections.

## Discussion

**Fig. 7:**
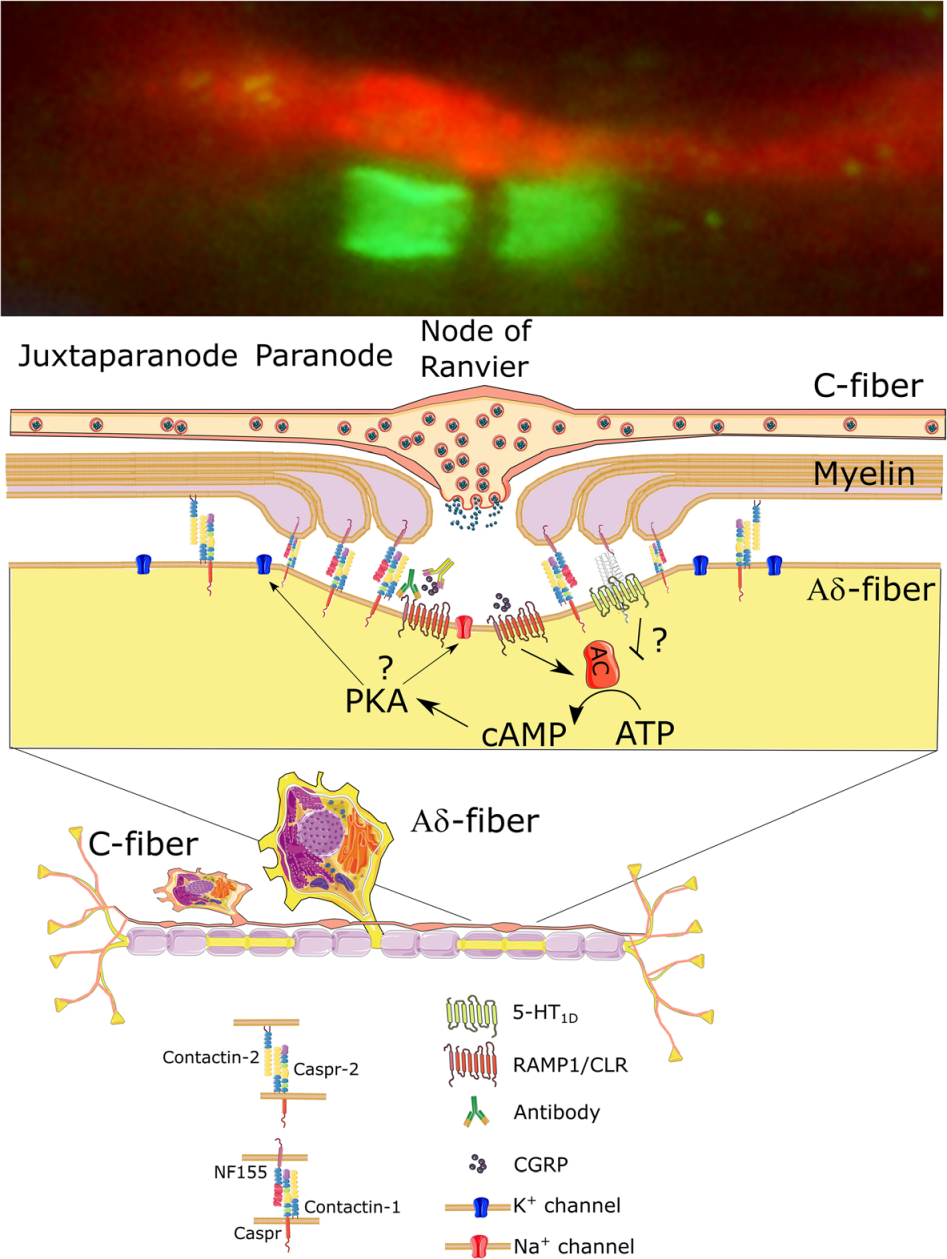
Schematic overview of the possible modulation by C-fibers on adjacent Aδ-fibers through axon-axon signaling at nodes of Ranvier in the trigeminal system. CGRP is released from the C-fiber bouton and diffuses into the node of Ranvier. Activation of the CGRP receptor causes increase in cyclic AMP, which could alter the conductivity of Na^+^ or K^+^ channels through Phosphokinase A (PKA). Furthermore, there is a possibility that 5-HT receptors could negatively modulate the cyclic AMP at the node. Alterations in Na^+^ or K^+^ channels could modulate the nodal threshold of activation. The schematic also includes a hypothesis on the mode of action of the anti-migraine antibody treatments

This is the first study to provide evidence for the innovative hypothesis that axon-axon CGRP signaling occurs between C-fibers and the Aδ-fibers in the TG and in the dura mater. These findings reveal a novel site where specific anti-migraine drugs may act to suppress trigeminal pain transmission. The use of a CASPR antibody allowed us to selectively visualize the nodes of Ranvier in sensory Aδ-fibers and revealed the key, but unexpected, finding that CGRP receptors co-localize with CASPR. The nodes of Ranvier are central to the process of salutatory conduction of axon potentials that underlies faster transmission in myelinated nerves [27]. Modulation of ion channels within the nodes could affect the threshold and rate of nerve firing. The CASPR antibody also revealed a specific pattern of nodes in close proximity to CGRP-positive boutons on C-fibers. Furthermore, we demonstrated that CGRP can be released from C-fibers in the TG. Together these data suggest that CGRP regulates transmission in trigeminal nerves via axon-axon signaling (Fig. 7). This process may be an important mechanism in the pathophysiology of migraine and may present a novel site of action for anti-migraine drugs.

### CGRP as an axonal signaling molecule

CGRP release has previously been demonstrated in the dura mater, the trigeminal nucleus caudalis (TNC) and neuronal cell bodies in the trigeminal vascular system [21, 28–30]. In the current study we show that CGRP can be released directly from trigeminal nerve fibers in response to depolarization. The thin, unmyelinated C-fibers display a “pearl-like” pattern of CGRP-containing varicosities at regular intervals along the nerve. These varicosities, also referred to as “en-passant boutons”, are characteristic of presynaptic release sites in peripheral nerves [31]. There is a close association of C- and Aδ-fibers within the TG, the trigeminal nerve and peripheral targets such as the dura mater that suggests possible CGRP signaling between the two types of pain fibers [9]. Remarkably, co-staining with CASPR and CGRP Abs revealed that CGRP-labeled varicosities align closely with nodes of Ranvier on Aδ-fibers that express CGRP receptors. These structures are suggestive of axon-axon synapses. To our knowledge, this mode of interaction has not been reported before.

### Possible mechanism of sensitization

The role of CGRP receptors located in the nodes of Ranvier of Aδ-fibers remains speculative, however it seems likely they are involved in modulating ion channels, sensitivity thresholds and/or nerve firing rates. The CGRP receptor couples to a transmembrane G-protein (Gα_s_) on the cytosolic side of the membrane. The Gα_s_ subunit activates adenylyl cyclase which in turn catalyzes the conversion of ATP into the second messenger cyclic AMP. Increasing cytosolic cyclic AMP concentration results in activation of protein kinase A (PKA), which we found in the nodal region, and other intracellular pathways [32, 33]. Through kinase activation, CGRP could regulate ion channels essential for nerve signal propagation [34–36] and contribute to mechanisms of neural sensitization and/or increased intensity of pain transmission in migraine. Nodes of Ranvier are complex, highly organized structures in which the central, unmyelinated portion of the node itself is flanked by paranodal and juxtaparanodal regions [15].

Gradation of the node and internode properties along axons can tune conduction speed and activation threshold [37]. This could occur by altering either the activity of excitatory sodium channels or inhibitory potassium channels. We focus here on excitatory activation. A possible mechanism of pain generation by K^+^-channels has been discussed elsewhere [12]. The node of Ranvier contains voltage-gated sodium channels (Na_V_) such as Na_V_1.6 and Na_V_1.1 [38], while voltage-gated potassium channels (K_V_) are localized to the juxtaparanodal region [39, 40].

Individual anatomical parameters of myelinated axons can be tuned to optimize pathways involved in temporal processing. For example, additional Na_V_ channels such as Na_V_1.7 (expressed at nodes of Ranvier in a subpopulation of Aδ-fibers within sciatic nerve and dorsal root ganglion, DRG) has been shown to modulate pain perception in animal models [41, 42]. These observations are consistent with critical roles for Na_V_1.7 channels at multiple sites within nociceptive DRG neurons and their processes [43]. It has further been suggested that during neuropathic pain (in the DRG), an increase in cyclic AMP would induce a negative shift of the activation of sodium currents. The action potential threshold would thus decrease and neuronal hyperexcitability would increase [44].

We postulate that similar activation by nodal CGRP receptors could occur in migraine patients.

In addition to being a potential target for the current drugs targeting the CGRP receptor or CGRP release, we questioned if other anti-migraine drugs also target axonal nodes and possible interactions of C- and Aδ-fibers. The current mechanism of action of triptans, ditans and potentially new anti-migraine compounds, e.g. agonists aimed at the P2Y_13_ receptor, have been linked to inhibiting CGRP release through receptors coupling to G_i_ proteins [12, 22, 45, 46]. In the current paper, we show an effect of triptans in TG regions that contain neuronal cell bodies, but not in fiber-rich areas devoid of somas. These data offer an explanation to why for example sumatriptan does not fully inhibit CGRP release in previous studies from the TG [30] but has a full effect on isolated TG neurons [29].

### Site of action for anti-migraine drugs

The effectiveness of mAbs targeting CGRP or its receptor indicates that their site of action must be in the periphery as the mAbs do not cross the BBB [5]. Triptans are also considered to have little to no ability to cross the BBB [47]. Moreover, there is no evidence that the BBB is altered during a migraine attack [6–8]. Currently, hypothesized drug targets include trigeminal neuron synapses, neuronal somas in the TG, or the synapses localized in the TNC [1, 2, 11, 12]. The role of Aδ-fibers in migraine pathology was recently strengthen by the work of Burstein and colleagues which concluded that dilation and plasma protein extravasation induced by cortical spreading depression are unaffected by the anti-CGRP mAb; Fremanezumab [10]. Therefore, the most likely target of CGRP released in migraine is the Aδ-fibers. The current study indicates a potential specific and novel target located on the Aδ-fiber, namely axo-axonal synapses between the C-fibers and Aδ-fibers and suggests that such interactions can occur along the sensory fibers, altering sensory processing.

The TG is outside the BBB [48, 49], however the question arises as to whether the nodes of Ranvier would be accessible to anti-migraine Abs. It has been shown that the node of Ranvier is indeed permeable to both smaller and larger water-soluble molecules. Miezwa and colleagues have investigated this in depth, using 3 kDa and 70 kDa dextran tracers coupled to fluorescein, which is comparable to the size of CGRP (5 kDa) and the size of the mAbs (150 kDa). Both 3 and 70 kDa tracers are able to penetrate from the perinodal space symmetrically into the paranodes on either side of the node of Ranvier at a rate consistent with diffusion through an elongated helical pathway between the paranodal terminal loops of the myelin sheath. Hence, it appears likely that CGRP receptors within the node of Ranvier are an accessible drug target [50].

### Conclusion and perspective

To our knowledge, this is the first study to provide evidence for axon-axon synapses between adjacent sensory nerve fibers. Use of an Ab to the nodal marker CASPR was instrumental in clarifying the relationship of nodal CGRP receptors in Aδ-fibers to CGRP release sites in varicosities in C-fibers. While more needs to be learned, the ability of CGRP to regulate the excitability of trigeminal fibers via the nodes of Ranvier would be a significant mechanism in modulating pain transmission. This mechanism would likely contribute to migraine headache pathology and provide a novel target for anti-migraine drugs.

## Supplementary information

**Additional file 1: Figure S1.** CASPR expression in relation to myelin basic protein. Immunolocalization of CASPR positive axonal nodes (arrow) in the trigeminal ganglion in relation to myelin basic protein (MBP)

**Additional file 2: Figure S2.** Early endosome autoantigen in the TG fibers. Immunolocalization of CASPR-positive axonal nodes (arrow) in the trigeminal ganglion in relation to axonally transported endosomes, labeled with an Ab to EEA1 (Early endosomal autoantigen 1)

**Additional file 3: Table S1.** Description of primary antibodies used in this study.

**Additional file 4: Table S2.** Description of secondary antibodies used in this study

## Data Availability

The datasets generated during and/or analyzed during the current study are available from the corresponding author on reasonable request.

## Abbreviations

5-HT: 5-Hydroxytryptamine
BBB: Blood-brain barrier
BSA: Bovine serum albumin
CAMP: Cyclic adenosine monophosphate
CASPR: Contactin associated protein
CGRP: Calcitonin gene-related peptide
CLR: Calcitonin like receptor
CNS: Central nervous system
DRG: Dorsal root ganglion
EEA1: Early endosomal antigen
MAbs: Monoclonal antibodies
PBS: Phosphate buffered saline
PKA: Protein kinase A
PNS: Peripheral nervous system
RAMP1: Receptor activity modifying protein
SIF: Synthetic interstitial fluid
TG: Trigeminal ganglion
TNC: Trigeminal nucleus caudalis

## References

[1] Edvinsson L, Haanes KA, Warfvinge K, Krause DN (2018). CGRP as the target of new migraine therapies - successful translation from bench to clinic. Nat Rev Neurol.

[2] Edvinsson L (2017). The Trigeminovascular pathway: role of CGRP and CGRP receptors in migraine. Headache..

[3] Olesen J, Diener HC, Husstedt IW, Goadsby PJ, Hall D, Meier U (2004). Calcitonin gene-related peptide receptor antagonist BIBN 4096 BS for the acute treatment of migraine. N Engl J Med.

[4] Lattanzi S, Brigo F, Trinka E, Vernieri F, Corradetti T, Dobran M (2019). Erenumab for preventive treatment of migraine: a systematic review and meta-analysis of efficacy and safety. Drugs..

[5] Boado RJ, Zhou QH, Lu JZ, Hui EK, Pardridge WM (2010). Pharmacokinetics and brain uptake of a genetically engineered bifunctional fusion antibody targeting the mouse transferrin receptor. Mol Pharm.

[6] Edvinsson L, Tfelt-Hansen P (2008). The blood-brain barrier in migraine treatment. Cephalalgia..

[7] Hougaard A, Amin FM, Christensen CE, Younis S, Wolfram F, Cramer SP (2017). Increased brainstem perfusion, but no blood-brain barrier disruption, during attacks of migraine with aura. Brain..

[8] Amin FM, Hougaard A, Cramer SP, Christensen CE, Wolfram F, Larsson HBW (2017). Intact blood-brain barrier during spontaneous attacks of migraine without aura: a 3T DCE-MRI study. Eur J Neurol.

[9] Eftekhari S, Warfvinge K, Blixt FW, Edvinsson L (2013). Differentiation of nerve fibers storing CGRP and CGRP receptors in the peripheral trigeminovascular system. J Pain.

[10] Schain AJ, Melo-Carrillo A, Stratton J, Strassman AM, Burstein R (2019). CSD-induced arterial dilatation and plasma protein extravasation are unaffected by Fremanezumab: implications for CGRP's role in migraine with Aura. J Neurosci.

[11] Melo-Carrillo A, Strassman AM, Nir RR, Schain AJ, Noseda R, Stratton J (2017). Fremanezumab-a humanized monoclonal anti-CGRP antibody-inhibits thinly Myelinated (Adelta) but not unmyelinated (C) meningeal Nociceptors. J Neurosci.

[12] Haanes KA, Edvinsson L (2019). Pathophysiological mechanisms in migraine and the identification of new therapeutic targets. CNS Drugs.

[13] Zhang L, Kunkler PE, Knopp KL, Oxford GS, Hurley JH (2019). Role of intraganglionic transmission in the trigeminovascular pathway. Mol Pain.

[14] Walker CS, Raddant AC, Woolley MJ, Russo AF, Hay DL (2018). CGRP receptor antagonist activity of olcegepant depends on the signalling pathway measured. Cephalalgia..

[15] Salzer JL (1997). Clustering sodium channels at the node of Ranvier: close encounters of the axon-glia kind. Neuron..

[16] Arancibia-Carcamo IL, Attwell D (2014). The node of Ranvier in CNS pathology. Acta Neuropathol.

[17] Arroyo EJ, Scherer SS (2000). On the molecular architecture of myelinated fibers. Histochem Cell Biol.

[18] Rios JC, Melendez-Vasquez CV, Einheber S, Lustig M, Grumet M, Hemperly J (2000). Contactin-associated protein (Caspr) and contactin form a complex that is targeted to the paranodal junctions during myelination. J Neurosci.

[19] Einheber S, Zanazzi G, Ching W, Scherer S, Milner TA, Peles E (1997). The axonal membrane protein Caspr, a homologue of neurexin IV, is a component of the septate-like paranodal junctions that assemble during myelination. J Cell Biol.

[20] Dreisig K, Blixt FW, Warfvinge K (2018) Retinal Cryo-sections, whole-mounts, and hypotonic isolated vasculature preparations for Immunohistochemical visualization of microvascular Pericytes. *JoVE* (140):e57733 10.3791/57733 PMC6235433 30346386

[21] Bhatt DK, Gupta S, Jansen-Olesen I, Andrews JS, Olesen J (2013). NXN-188, a selective nNOS inhibitor and a 5-HT1B/1D receptor agonist, inhibits CGRP release in preclinical migraine models. Cephalalgia..

[22] Haanes Kristian A, Labastida-Ramírez Alejandro, Blixt Frank W, Rubio-Beltrán Eloisa, Dirven Clemens M, Danser Alexander HJ, Edvinsson Lars, MaassenVanDenBrink Antoinette (2019). Exploration of purinergic receptors as potential anti-migraine targets using established pre-clinical migraine models. Cephalalgia.

[23] Eftekhari S, Edvinsson L (2011). Calcitonin gene-related peptide (CGRP) and its receptor components in human and rat spinal trigeminal nucleus and spinal cord at C1-level. BMC Neurosci.

[24] Miller S, Liu H, Warfvinge K, Shi L, Dovlatyan M, Xu C (2016). Immunohistochemical localization of the calcitonin gene-related peptide binding site in the primate trigeminovascular system using functional antagonist antibodies. Neuroscience..

[25] Cottrell GS (2018) CGRP receptor Signalling pathways. Handb Exp Pharmacol 10.1007/164_2018_130 30151722

[26] Jarnaess E, Tasken K (2007). Spatiotemporal control of cAMP signalling processes by anchored signalling complexes. Biochem Soc Trans.

[27] Ghosh A, Sherman DL, Brophy PJ (2018). The axonal cytoskeleton and the assembly of nodes of Ranvier. Neuroscientist..

[28] Ebersberger A, Averbeck B, Messlinger K, Reeh PW (1999). Release of substance P, calcitonin gene-related peptide and prostaglandin E2 from rat dura mater encephali following electrical and chemical stimulation in vitro. Neuroscience..

[29] Durham PL, Russo AF (1999). Regulation of calcitonin gene-related peptide secretion by a serotonergic antimigraine drug. J Neurosci.

[30] Amrutkar DV, Ploug KB, Hay-Schmidt A, Porreca F, Olesen J, Jansen-Olesen I (2012). mRNA expression of 5-hydroxytryptamine 1B, 1D, and 1F receptors and their role in controlling the release of calcitonin gene-related peptide in the rat trigeminovascular system. Pain..

[31] Smolen AJ (1988). Morphology of synapses in the autonomic nervous system. J Electron Microsc Tech.

[32] Egea SC, Dickerson IM (2012). Direct interactions between calcitonin-like receptor (CLR) and CGRP-receptor component protein (RCP) regulate CGRP receptor signaling. Endocrinology..

[33] Russell FA, King R, Smillie SJ, Kodji X, Brain SD (2014). Calcitonin gene-related peptide: physiology and pathophysiology. Physiol Rev.

[34] Sassone-Corsi P. (2012). The Cyclic AMP Pathway. Cold Spring Harbor Perspectives in Biology.

[35] Scheuer T (2011). Regulation of sodium channel activity by phosphorylation. Semin Cell Dev Biol.

[36] Liu S, Zheng P (2013). Altered PKA modulation in the Nav1.1 epilepsy variant I1656M. J Neurophysiol.

[37] Arancibia-Carcamo IL, Ford MC, Cossell L, Ishida K, Tohyama K, Attwell D (2017) Node of Ranvier length as a potential regulator of myelinated axon conduction speed. Elife. 6 10.7554/eLife.23329 PMC5313058 28130923

[38] Shrager P (1989). Sodium channels in single demyelinated mammalian axons. Brain Res.

[39] Wang H, Kunkel DD, Martin TM, Schwartzkroin PA, Tempel BL (1993). Heteromultimeric K+ channels in terminal and juxtaparanodal regions of neurons. Nature..

[40] Mi H, Deerinck TJ, Ellisman MH, Schwarz TL (1995). Differential distribution of closely related potassium channels in rat Schwann cells. J Neurosci.

[41] Lee JH, Park CK, Chen G, Han Q, Xie RG, Liu T (2014). A monoclonal antibody that targets a NaV1.7 channel voltage sensor for pain and itch relief. Cell..

[42] Yang S, Xiao Y, Kang D, Liu J, Li Y, Undheim EA (2013). Discovery of a selective NaV1.7 inhibitor from centipede venom with analgesic efficacy exceeding morphine in rodent pain models. Proc Natl Acad Sci U S A.

[43] Black JA, Frézel N, Dib-Hajj SD, Waxman SG (2012). Expression of Nav1.7 in DRG neurons extends from peripheral terminals in the skin to central preterminal branches and terminals in the dorsal horn. Mol Pain.

[44] Chatelier A, Dahllund L, Eriksson A, Krupp J, Chahine M (2008). Biophysical properties of human Na v1.7 splice variants and their regulation by protein kinase a. J Neurophysiol.

[45] Humphrey PP (2007). The discovery of a new drug class for the acute treatment of migraine. Headache..

[46] Rubio-Beltrán E, Labastida-Ramírez A, Haanes KA, Bogaerdt A, Bogers AJJC, Zanelli E, Meeus L, Danser AHJ, Gralinski MR, Senese PB, Johnson KW, Kovalchin J, Villalón CM, MaassenVanDenBrink A (2019) Characterization of binding, functional activity and contractile responses of the selective 5-HT receptor agonist lasmiditan. British Journal of Pharmacology 10.1111/bph.14832 PMC6965684 31418454

[47] Pascual J, Munoz P (2005). Correlation between lipophilicity and triptan outcomes. Headache..

[48] Lundblad C, Haanes KA, Grande G, Edvinsson L. (2015) Experimental inflammation following dural application of complete Freund's adjuvant or inflammatory soup does not alter brain and trigeminal microvascular passage. *The journal of headache and pain*. 16:91 10.1186/s10194-015-0575-8 PMC4627622 26512021

[49] Eftekhari S, Gaspar RC, Roberts R, Chen TB, Zeng Z, Villarreal S (2016). Localization of CGRP receptor components and receptor binding sites in rhesus monkey brainstem: a detailed study using in situ hybridization, immunofluorescence, and autoradiography. J Comp Neurol.

[50] Mierzwa A, Shroff S, Rosenbluth J (2010). Permeability of the paranodal junction of myelinated nerve fibers. J Neurosci.

