# Supplementary figures and images for: C-fibers may modulate adjacent Aδ-fibers through axon-axon CGRP signaling at nodes of Ranvier in the trigeminal system
Source: J Headache Pain. 2019 Nov 12;20(1):105. doi: 10.1186/s10194-019-1055-3 (PMC6852900; doi:10.1186/s10194-019-1055-3)

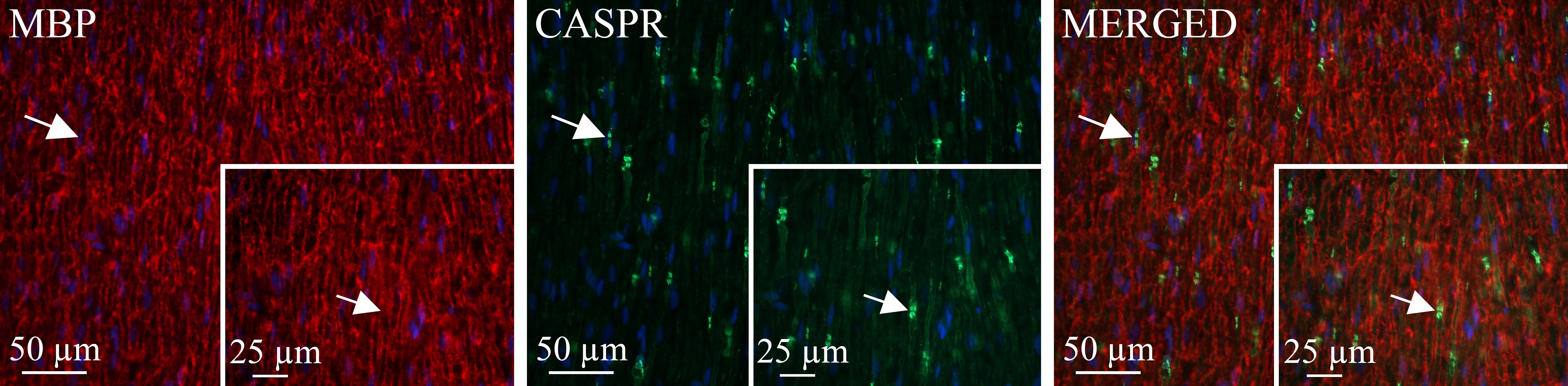

Supplement: Supplementary file 1 — Additional file 1: Figure S1. CASPR expression in relation to myelin basic protein. Immunolocalization of CASPR positive axonal nodes (arrow) in the trigeminal ganglion in relation to myelin basic protein (MBP) [file 10194_2019_1055_MOESM1_ESM.jpg]

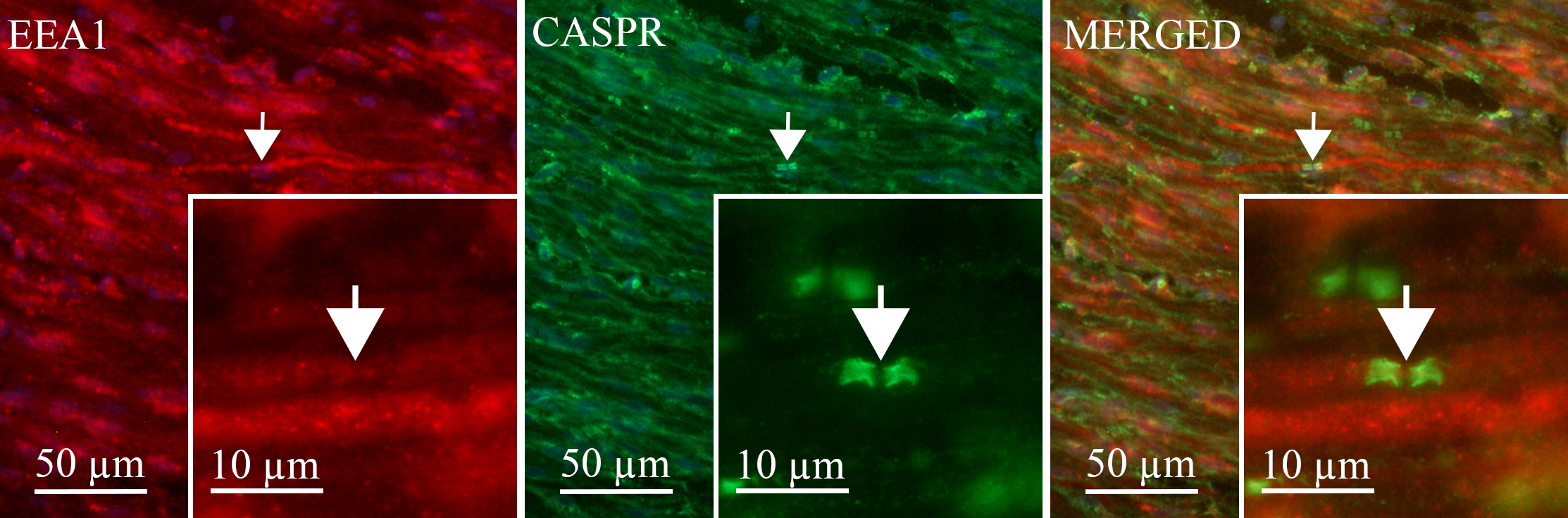

Supplement: Supplementary file 2 — Additional file 2: Figure S2. Early endosome autoantigen in the TG fibers. Immunolocalization of CASPR-positive axonal nodes (arrow) in the trigeminal ganglion in relation to axonally transported endosomes, labeled with an Ab to EEA1 (Early endosomal autoantigen 1) [file 10194_2019_1055_MOESM2_ESM.png]
