# Supplementary material for: C-fibers may modulate adjacent Aδ-fibers through axon-axon CGRP signaling at nodes of Ranvier in the trigeminal system
Source: J Headache Pain. 2019 Nov 12;20(1):105. doi: 10.1186/s10194-019-1055-3 (PMC6852900; doi:10.1186/s10194-019-1055-3)
Supplement: Supplementary file 3 — Additional file 3: Table S1. Description of primary antibodies used in this study. [file 10194_2019_1055_MOESM3_ESM.docx]

Supplementary table 1

| Primary antibodies | | | | | | |
| --- | --- | --- | --- | --- | --- | --- |
| Name | Cat# | Host | Clonality | Dilution | Immunogen | Source |
| Anti-CASPR, clone K65/35 | MABN69 | Mouse | Monoclonal | 1:100 | Recombinant protein corresponding to rat Caspr. | EMD Millipore Corporation, Temecula, CA, USA. |
| Anti-CASPR | AB34151 | Rabbit | Polyclonal | 1:250 | Synthetic peptide conjugated to keyhole limpet hemocyanin derived from within residues 1350 to the C-terminus of Mouse CASPR. | Abcam, Cambridge, UK |
| Anti-PKA C-α Antibody | #4782 | Rabbit | Polyclonal | 1:100 | Synthetic peptide derived from the carboxy terminal sequence of human PKA C-α. | Cell Signaling Technology, Danvers, MA, USA. |
| Anti-CGRP antibody | ab81887 | Mouse | Monoclonal | 1:100 | Rat alpha-CGRP | Abcam, Cambridge, UK |
| Anti-MBP antibody | MA5–15922 | Mouse | Monoclonal | 1:100 | Purified recombinant fragment of human MBP expressed in *E. coli*. | Thermo Fisher Scientific, Waltham, MA, USA |
| AA58 | – | Human | Monoclonal | 20 µg/mL | [24] | Amgen Inc., Thousand Oaks, CA, USA |
| Anti-RAMP1 | 844 | Goat | Monoclonal | 1:200 | C-terminal of human RAMP1 | Merck & Co, Inc., West Point, PA, USA |
| Anti-EEA1 | #3288 | Rabbit | Monoclonal | 1:100 | Synthetic peptide corresponding to residues surrounding Ser70 of human EEA1 protein | Cell Signaling Technology, Danvers, MA, USA |
