# Supplementary material for: C-fibers may modulate adjacent Aδ-fibers through axon-axon CGRP signaling at nodes of Ranvier in the trigeminal system
Source: J Headache Pain. 2019 Nov 12;20(1):105. doi: 10.1186/s10194-019-1055-3 (PMC6852900; doi:10.1186/s10194-019-1055-3)
Supplement: Supplementary file 4 — Additional file 4: Table S2. Description of secondary antibodies used in this study [file 10194_2019_1055_MOESM4_ESM.docx]

Supplementary table. 2

| Secondary antibodies | | | |
| --- | --- | --- | --- |
| Name | Dilution | Against | Source |
| Alexa Fluor 594 | 1:100 | Anti-Human | Thermo Fisher Scientific, MA, USA |
| FITC | 1:100 | Anti-Rabbit | Cayman Chemical, Ann Arbor, MI, USA |
| Alexa Fluor 594 | 1:100 | Anti-Rabbit | Jackson Immunoresearch Laboratories, Inc., West Grove, PA, USA |
| Alexa Fluor 594 | 1:100 | Anti-Mouse | Invitrogen, CA, USA |
| FITC | 1:100 | Anti-Mouse | Jackson Immunoresearch Laboratories, Inc., West Grove, PA, USA |
| Cy3 | 1:400 | Anti-Goat | Jackson Immunoresearch Laboratories, Inc., West Grove, PA, USA |
